# Epithelial cell-fate switch triggering ectopic ligand-receptor-mediated JAK-STAT signaling promotes tumorigenesis in *Drosophila*

**DOI:** 10.1016/j.isci.2025.112191

**Published:** 2025-03-10

**Authors:** Jiaqi Li, Kiichiro Taniguchi, Weiran Ye, Shu Kondo, Tomoe Kobayashi, Makoto Matsuyama, Kuniaki Saito, Shizue Ohsawa, Tatsushi Igaki

**Affiliations:** 1 Laboratory of Genetics, Graduate School of Biostudies, Kyoto University, Yoshida-Konoe-cho, Sakyo-ku, Kyoto 607-8501, Japan; 2 Department of Biological Science and Technology, Faculty of Advanced Engineering, Tokyo University of Science, 6-3-1 Niijuku, Katsushika-ku, Tokyo 125-8585, Japan; 3 Division of Molecular Genetics, Shigei Medical Research Institute, 2117, Minami-ku, Yamada, Okayama 701-0202, Japan; 4 Invertebrate Genetics Laboratory, National Institute of Genetics, 1111 Yata, Mishima, Shizuoka 411-8540, Japan; 5 Laboratory of Genetics, Graduate School of Science, Nagoya University, Furo-cho, Chikusa-ku, Nagoya, Aichi 464-8602, Japan

**Keywords:** Cell biology, Organizational aspects of cell biology, Cancer

## Abstract

Disruption of epithelial architecture is a hallmark of human malignant cancers, yet whether and how epithelial deformation influences tumor progression has been elusive. Here, through a genetic screen in *Drosophila* eye disc, we explored mutations that potently promoted Ras-activated (Ras^V12^) tumor growth and identified *eyes absent* (*eya*), an eye determination gene, whose mutation compromised tissue growth but synergized with Ras^V12^ to cause massive overgrowth. Furthermore, induction of cell-fate switch by mis-expression of *Abd-B* in the eye disc also induced massive Ras^V12^ overgrowth. Mechanistically, cell-fate switch caused epithelial invagination accompanied by partial mislocalization of the transmembrane receptor Domeless (Dome) from the apical to the basal membrane of the eye epithelium, where its ligand Unpaired3 (Upd3) is present. This led to JAK-STAT activation that cooperates with Ras^V12^ to drive tumor progression. Our data provide a mechanistic explanation for how cell-fate switch and subsequent epithelial deformation creates a cancer-prone environment in the epithelium.

## Highlights

• Cell-fate switch promotes tumorigenesis in *Drosophila* epithelium
• JAK-STAT signaling is activated in cells with cell-specification defect
• Cell-fate switch causes mislocalization of a transmembrane receptor Domeless
• Mislocalized Domeless interacts with its ligand Upd3, thereby activating JAK-STAT

## Graphical abstract

**Figure:**
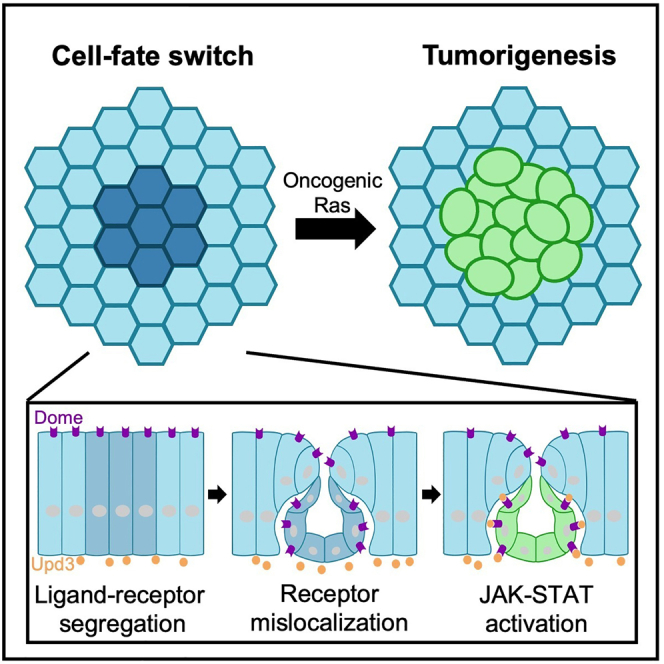

## Introduction

Gain-of-function mutations of the Ras family genes are frequently observed in human cancers, yet a comprehensive understanding of the molecular circuitries underlying Ras-induced tumorigenesis is still missing. Oncogenic Ras activation alone has limited tumorigenic ability itself,^1^^,^^2^ suggesting that additional mutations or cellular changes drive tumor progression of Ras-activated cells. In *Drosophila*, oncogenic Ras (Ras^V12^) causes benign overgrowths, while additional deficiency of apico-basal polarity genes strongly enhances tumor growth and invasion.^3^^,^^4^ Other cellular alterations, such as reduced calcium concentration in endoplasmic reticulum (ER),^5^ defects in tri-cellular junction,^6^ autophagy inhibition,^7^ and senescence evasion,^8^ also cooperate with Ras^V12^ to drive tumor progression. Despite intensive exploration, a common pathophysiological condition orchestrating progression of Ras-activated benign tumors is still unknown.

Tumor entities often display disruption of tissue architecture and modification of mechanical microenvironment, which may cause genetic, epigenetic, and phenotypic effects on cancer cells.^9^ Carcinomas often form buds or folds before becoming aggressive, and Ras/MAPK-driven squamous cell carcinoma or colorectal carcinoma display apical invagination and tissue folding at the onset of carcinogenesis.^10^^,^^11^ In *Drosophila*, neoplasia prefers to arise at tumor “hot-spots” in the epithelium with abundant curvatures.^12^ However, whether and how epithelial deformation influences tumor progression has been elusive.

Here, through a genetic screen in *Drosophila* eye discs using a CRISPR-Cas9-based knockout fly library, we isolate mutations in *eyes absent* (*eya*), an eye specification gene, that cooperate with Ras^V12^ to strongly promote tumorigenesis. Loss of *eya* induces epithelial invagination, which causes activation of JAK-STAT signaling, thereby synergizing with Ras^V12^ to boost tumor growth. Mechanistically, epithelial deformation caused by *eya* mutation triggers partial mislocalization of the transmembrane receptor Domeless (Dome) from the apical to basal membrane of the eye epithelium, which allows Dome to meet its ligand Unpaired3 (Upd3, an IL-6 homolog), thereby activating downstream JAK-STAT signaling. Our data propose that cell-fate switch and resulting epithelial deformation act as driving forces of Ras-activated tumorigenesis by inducing interaction of normally segregated oncogenic ligand and receptor in the epithelium.

## Results

### Cell-fate switch cooperates with Ras to strongly enhance tumorigenesis

**Figure 1:**
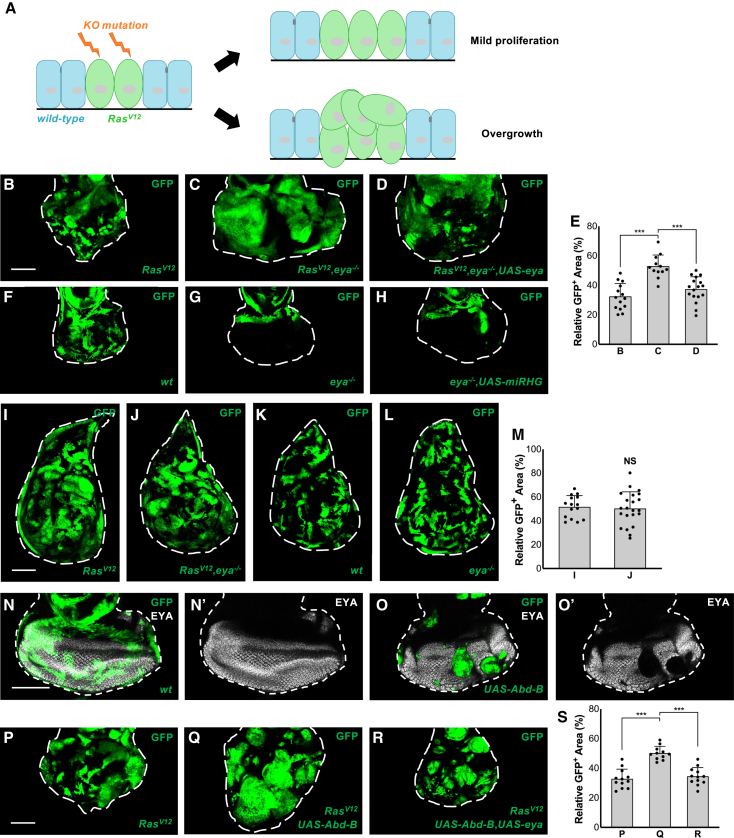
*eya* mutation enhances Ras^V12^-induced tumorigenesis in the eye epithelium (A) Screening scheme to identify knock-out mutations that promote the overgrowth of Ras^V12^ clones in eye imaginal epithelium using *eyFLP* MARCM technique by GFP-labeled clone size. Ras^V12^ clones proliferate moderately, while introducing further mutations could lead to strong overgrowth. (B–D) Eye discs bearing GFP-labeled MARCM clones of Ras^V12^ (B), Ras^V12^+*eya*^−/−^ (C), or Ras^V12^ +*eya*^−/−^+UAS-*eya* (D). Scale bar: 100 μm, and applicable for (F)–(H). (E) Quantification of relative GFP clone size (% of total clone area/disc area in the eye disc) for (B)–(D) (*n* > 10, number of eye discs). ∗∗∗*p* < 0.001; Kruskal-Wallis test. Data are represented as mean ± SD. (F–H) Eye discs bearing GFP-labeled MARCM clones of wild-type (F), *eya*^−/−^ (G), or *eya*^−/−^+UAS-*miRHG* (H). (I–L) Wing discs bearing GFP-labeled MARCM clones of Ras^V12^ (I), Ras^V12^+*eya*^−/−^ (J), wild-type (K), or *eya*^−/−^ (L). Scale bar: 100 μm (M) Quantification of GFP clone size for (I and J) (*n* > 10, number of eye discs). Mann-Whitney test. Data are represented as mean ± SD. (N and O) Eye discs bearing GFP-labeled MARCM clones of wild-type (I), or UAS-*Abd-B* (J), stained with anti EYA (white). Scale bar: 100 μm (P–R) Eye discs bearing GFP-labeled MARCM clones of Ras^V12^ (P), Ras^V12^+UAS-*Abd-B* (Q), or Ras^V12^ +UAS-*Abd-B*+UAS-*eya* (R). Scale bar: 100 μm (S) Quantification of relative GFP clone size for (P)–(R) (*n* > 10, number of eye discs). ∗∗∗*p* < 0.001; Kruskal-Wallis test. Data are represented as mean ± SD.

To identify mutations that potently promote Ras-induced tumorigenesis, a series of CRISPR-Cas9-mediated knockout mutations (see Materials and Methods) were introduced in GFP-labeled Ras^V12^-overexpressing clones using the Flippase (FLP)-FLP recognition target (FRT)-mediated genetic mosaic technique (mosaic analysis with a repressible cell marker, MARCM) in *Drosophila* eye-antennal discs (Figure 1A).^13^ We screened more than 1,400 mutant lines for genes in chromosome 2L and identified two independent *eya* null alleles (Table S1) that significantly promoted growth of Ras^V12^ clones (Figures 1B and 1C, quantified in 1E). *eya* encodes a transcriptional cofactor essential for retinal cell specification.^14^ The overgrowth of Ras^V12^/*eya*^−/−^ clones was canceled by exogenous overexpression of EYA (Figure 1D, quantified in 1E), confirming that loss of *eya* promotes Ras-induced tumorigenesis. Notably, *eya*^−/−^ clones did not survive in the eye disc, a posterior part of the eye-antennal disc (Figure 1G, compare to wild-type clones in 1F), indicating that the overgrowth was caused by oncogenic cooperation between *eya* mutation and Ras^V12^. It has previously been shown that undifferentiated cells have greater potential to develop into aggressive tumors following oncogenic transformation.^15^ To rule out the possibility that apoptosis evasion rendered by Ras^V12^ caused *eya*^−/−^ clone overgrowth,^16^ microRNA for the pro-apoptotic genes *reaper*, *hid*, and *grim* (miRHG) was overexpressed in *eya*^−/−^ clones, which showed limited rescue effect (Figure 1H). In addition, ectopic expression of *Drosophila* inhibitor of apoptosis protein (Diap1) or baculorvirus protein p35, two strong caspase inhibitors,^17^ also failed to induce *eya*^−/−^ clone overgrowth compared with the Ras^V12^ combination (Figure S1). These results suggest that loss of cell specification on its own does not equip cells with higher proliferative propensity. To determine if the oncogenic cooperation is due to loss of eye-cell fate in the eye disc, we generated these mutant clones in the wing imaginal discs. *eya* mutant clones survived in the wing disc and did not promote Ras^V12^ overgrowth (Figures 1I–1L, quantified in 1M). These data indicate that *eya* mutation cooperates with Ras^V12^ to promote tumorigenesis via cell-fate switch in the eye epithelium.

We next questioned if cell-fate switch is a common enhancer for Ras^V12^-induced tumorigenesis. We thus overexpressed selector genes for other tissues such as *Abdominal B* (*Abd-B*), a gene required for determining abdominal segment patterning,^18^ in clones of cells in the eye disc to override the fate determination process of eye cells. *Abd-B*-overexpressing clones were unable to be specified as eye cells, as indicated by loss of EYA (Figure 1O, compare to 1N), mimicking *eya* mutant clones. Notably, *Abd-B* overexpression significantly enhanced the overgrowth of Ras^V12^ clones, which was canceled by restoring *eya* expression (Figures 1P–1R, quantified in 1S). Similar results were obtained using another wing cell fate determinant *vestigial* (*vg*), which indeed caused massive overgrowth with Ras^V12^ when overexpressed in the eye discs (Figures S2A and S2B). In addition, overexpression of different tissue determinants such as *Abd-B* and *eyeless* (*ey*) also cooperated with Ras^V12^ in the wing discs (Figures S2C–S2E). In line with our results, previous study has shown that ectopic misexpression of selector genes transforms polarity-deficient cell clones into neoplastic ones.^19^ Collectively, these data indicate that cell-fate switch generally has a tumor-promoting function with Ras activation.

### *eya* mutation cooperates with Ras^V12^ via JAK-STAT activation

We next aimed to dissect the mechanism of how *eya* mutation cooperates with Ras^V12^ to cause tumor overgrowth. A major driver of tumor growth in various *Drosophila* tumor models is the loss of cell polarity and activation of c-Jun N-terminal kinase (JNK) signaling.^3^^,^^20^^,^^21^ However, the localization of two cell polarity markers, atypical protein kinase (aPKC) and Crumbs (Crb), remained intact in *eya*^−/−^ clones (Figures S3A and S3B), and JNK phosphatase *puckered* (*puc*), a downstream effector of JNK signaling, was not upregulated in Ras^V12^/*eya*^−/−^ tumors (Figures S3C and S3D), suggesting that JNK is not involved in this oncogenic cooperation.

**Figure 2:**
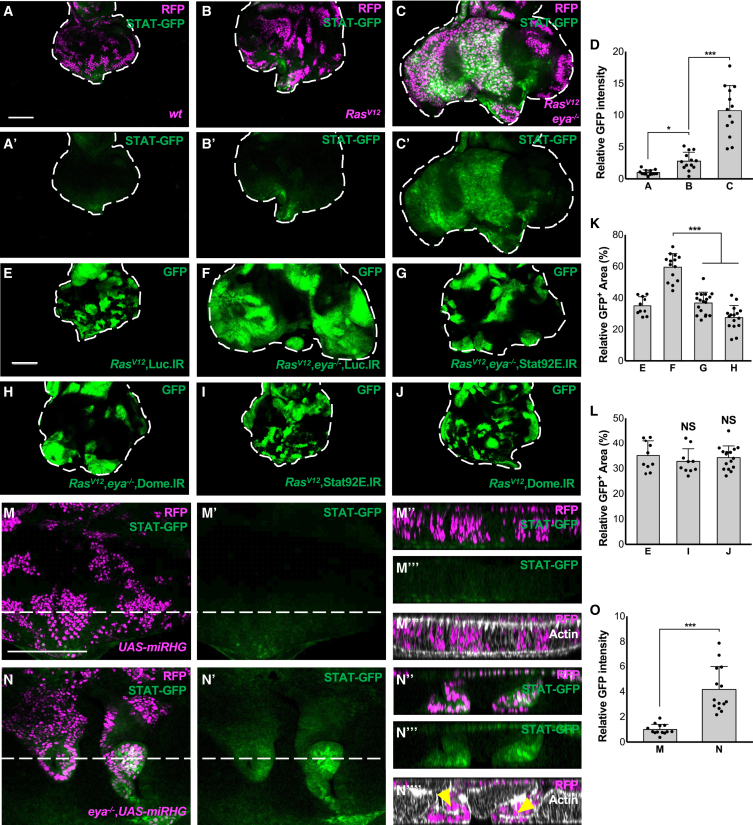
*eya* mutation activates JAK-STAT signaling, which cooperates with Ras^V12^ to induce tumor overgrowth (A–C) STAT-GFP expression in eye discs bearing RFP-labeled MARCM clones of wild-type (A), Ras^V12^ (B), or Ras^V12^+*eya*^−/−^ (C). Scale bar: 100 μm (D) Quantification of STAT-GFP intensity (average of A is set to 1) for (A)–(C) (*n* > 10, number of eye discs). ∗*p* < 0.1; ∗∗∗*p* < 0.001; Kruskal-Wallis test. Data are represented as mean ± SD. (E–J) Eye discs bearing GFP-labeled MARCM clones of Ras^V12^+Luc.IR (E), Ras^V12^+*eya*^−/−^ + Luc.IR (F), Ras^V12^+*eya*^−/−^ + Stat92E.IR (G), Ras^V12^+*eya*^−/−^ + Dome.IR (H), Ras^V12^+Stat92E.IR (J), or Ras^V12^+Dome. IR (K). Scale bar: 100 μm (K) Quantification of relative GFP clone size for (E)–(H) (*n* > 10, number of eye discs). ∗∗∗*p* < 0.001; Kruskal-Wallis test. Data are represented as mean ± SD. (L) Quantification of relative GFP clone size for (I) and (J) (*n* > 10, number of eye discs). Kruskal-Wallis test. Data are represented as mean ± SD. (M and N) STAT-GFP expression in eye discs bearing RFP-labeled MARCM clones of UAS-*miRHG* (M), or *eya*^−/−^+UAS-*miRHG* (N), stained with Phalloidin (white). Lines represent the position of lateral section images (M″–M‴ for M, N″–N‴ for N). Apical side to the top, basal side to the bottom. Arrowheads indicate accumulation of apical actin. Scale bar: 100 μm (O) Quantification of STAT-GFP intensity (average of M is set to 1) for (M) and (N) (*n* > 10, number of eye discs).∗∗∗*p* < 0.001; Mann-Whitney test. Data are represented as mean ± SD.

We searched for other pro-growth signaling activated in Ras^V12^/*eya*^−/−^ tumors and found that JAK-STAT signaling was significantly elevated in Ras^V12^/*eya*^−/−^, but not Ras^V12^, clones as visualized by the 10xStat92E-GFP (STAT-GFP) reporter (Figures 2A–2C, quantified in 2D). The JAK-STAT pathway is a prevalent tumor-promoting signaling in both mammals and flies,^20^^,^^21^^,^^22^ and when activated, could cooperate with Ras^V12^ to induce malignant overgrowth in *Drosophila* epithelium.^23^^,^^24^ Indeed, growth of Ras^V12^/*eya*^−/−^ tumors was significantly suppressed by knockdown of Stat92E (an STAT homolog) or Dome, a cell surface receptor that activates JAK-STAT signaling upon binding its ligand Upd, in these clones (Figures 2E–2H, quantified in 2K), while these knockdowns did not affect growth of Ras^V12^ clones (Figures 2I and 2J, quantified in 2L). Similarly, STAT-GFP was upregulated in Ras^V12^+*Abd-B* tumors (Figure S4A, quantified in Figure S4B) and this overgrowth was blocked by knockdown of Stat92E or Dome (Figures S4C and S4D, quantified in Figure S4E). Moreover, we found that STAT-GFP signal was elevated in *eya*^−/−^ clones (Figure 2N, miRHG was co-expressed to prevent cell death, compare to Figure 2M). Notably, *eya*^−/−^ clones displayed a deformed epithelial structure, characterized by epithelial invagination and accumulation of apical actin cytoskeleton (Figure 2N‴, compare to 2M‴). Consistently, it has been reported that mis-specified cell clones deform epithelial architecture by inducing invagination and cyst-like structures, which is considered to be an intrinsic tissue surveillance system to eliminate abnormally specified cells and could become precancerous lesions for oncogenic outgrowth.^19^^,^^25^^,^^26^ These data suggest that JAK-STAT activation caused by *eya*^−/−^ mutation cooperates with Ras^V12^ to induce tumor overgrowth.

### *eya* clones activate Dome-JAK-STAT signaling by extra-clonal Upd3

**Figure 3:**
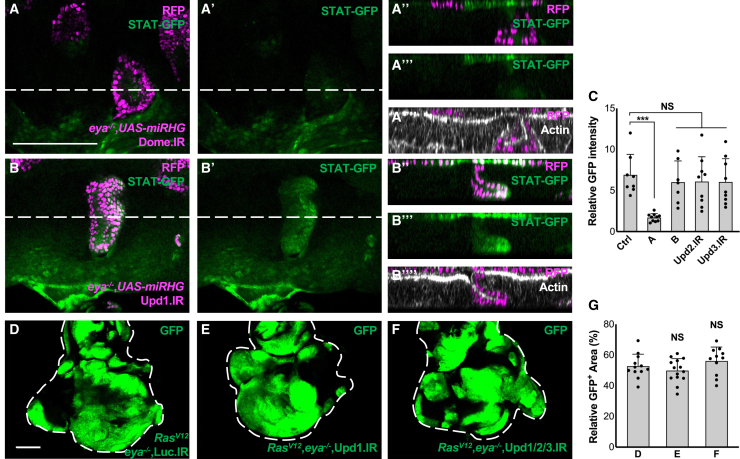
*eya* mutant clones activate Dome-JAK-STAT signaling independent of autonomous *upd* regulation (A and B) STAT-GFP expression in eye discs bearing RFP-labeled MARCM clones of *eya*^−/−^+UAS-*miRHG*+Dome.IR (A), or *eya*^−/−^+UAS-*miRHG*+Upd1.IR (B), stained with Phalloidin (white). Lines represent the position of lateral section images (A″-A‴ for A, B″-B‴ for B). Apical side to the top, basal side to the bottom. Scale bar: 100 μm (C) Quantification of STAT-GFP intensity for (A and B), compared with *eya*^−/−^+UAS-*miRHG* in Figure 2 (*n* > 6, number of eye discs). See also Figure S5. ∗∗∗*p* < 0.001; Kruskal-Wallis test. Data are represented as mean ± SD. (D–F) Eye discs bearing GFP-labeled MARCM clones of Ras^V12^+*eya*^−/−^ + Luc.IR (D), Ras^V12^+*eya*^−/−^ + Upd1.IR (E), or Ras^V12^+*eya*^−/−^ + Upd1.IR + Upd2.IR + Upd3.IR (F). Scale bar: 100 μm (G) Quantification of relative GFP clone size for (D)–(F) (*n* > 10, number of eye discs). Kruskal-Wallis test. Data are represented as mean ± SD.

In *Drosophila*, JAK-STAT signaling can be activated by the ligand Upd1, Upd2, or Upd3 via binding to their receptor Dome.^22^ Indeed, knockdown of Dome in *eya*^−/−^ clones blocked their STAT activation (Figure 3A, quantified in 3C). Intriguingly, knockdown of Upd1, Upd2, or Upd3 in *eya*^−/−^ clones did not suppress STAT activation (Figures 3B and S5A and S5B, quantified in 3C). Similarly, knockdown of Upds in Ras^V12^/*eya*^−/−^ clones did not suppress their growth (Figures 3D–3F, quantified in 3G). In addition, expression of *upd1* or *upd3*, which are known to promote imaginal disc growth,^27^ was not upregulated in *eya*^−/−^ clones, as visualized by the *upd1-lacZ* or *upd3-lacZ* reporter (Figures S5C–S5F). These data suggest that the ligand Upd is not derived from *eya*^−/−^ clones but from other cells.

**Figure 4:**
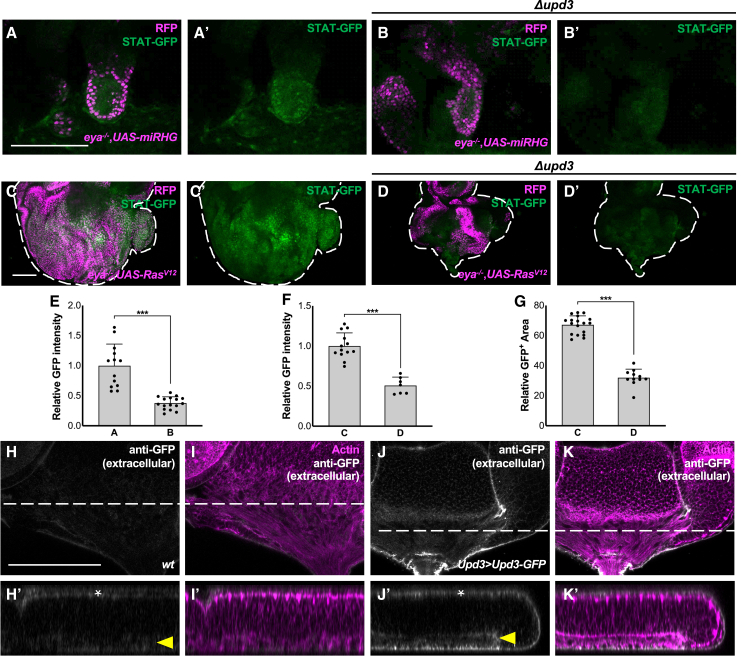
Upd3 is required for STAT activation and localized underneath the epithelial sheet (A and B) STAT-GFP expression in eye discs bearing RFP-labeled MARCM clones of *eya*^−/−^+UAS-*miRHG* (A), or *eya*^−/−^+UAS-*miRHG* with *Δupd3* background (B). Scale bar: 100 μm (C and D) STAT-GFP expression in eye discs bearing RFP-labeled MARCM clones of Ras^V12^+*eya*^−/−^(C), or Ras^V12^+*eya*^−/−^ with *Δupd3* background (D). Scale bar: 100 μm (E) Quantification of STAT-GFP intensity for (A and B) (*n* > 10, number of eye discs). ∗∗∗*p* < 0.001; Mann-Whitney test. Data are represented as mean ± SD. (F) Quantification of STAT-GFP intensity for (C and D) (*n* > 10, number of eye discs). ∗∗∗*p* < 0.001; Mann-Whitney test. Data are represented as mean ± SD. (G) Quantification of relative GFP clone size for (C and D) (*n* > 10, number of eye discs). ∗∗∗*p* < 0.001; Mann-Whitney test. Data are represented as mean ± SD. (H–K) Extracellular Upd3 distribution in eye discs of wild-type (H), or Upd3-Gal4+UAS-Upd3-GFP (J), stained with anti-GFP (white) and phalloidin (magenta). Lines represent the position of lateral section images (H′–K′ for H–K). Apical side to the top, basal side to the bottom. Asterisk indicates apical side, while arrowheads indicate basal side. Scale bar: 100 μm.

Recent studies highlight the systematic regulation of tumorigenesis through circulating molecules, such as insulin peptides and intestinal metabolites.^28^^,^^29^ We thus questioned if JAK-STAT activation was achieved by systemic Upd ligands. Notably, STAT-GFP intensity in *eya* mutant clones was significantly reduced in the eye discs of the larvae homozygously mutant for *upd3* (Figure 4B, compare to 4A, quantified in 4E). Upd3 deletion also reduced JAK-STAT signaling and thus tissue growth in Ras^V12^/*eya*^−/−^ clones (Figure 4D, compare to 4C, quantified in 4F and 4G). This suggests that the Dome-JAK-STAT signaling is activated in a ligand, Upd3-dependent manner. To explore the source of Upd3, we analyzed the localizations of Upd3 using UAS-Upd3-GFP in conjunction with *upd3*-Gal4.^27^ Immunostaining of extracellular GFP showed that Upd3 localized underneath the epithelial sheet (Figure 4J, compare to 4H, yellow arrowheads). Given that Upd3 is known to be expressed by glial cells that are located at the base of eye epithelium,^30^ the ligand for activating JAK-STAT signaling in *eya*^−/−^ clones could come from the basal extracellular region of the eye disc. It could also be possible that circulating Upd3 produced by immune cells is a source of the basal extracellular Upd3.^31^^,^^32^

### Cell-fate switch causes Upd3-Dome interaction via mislocalization of Dome from the apical to basal membrane

**Figure 5:**
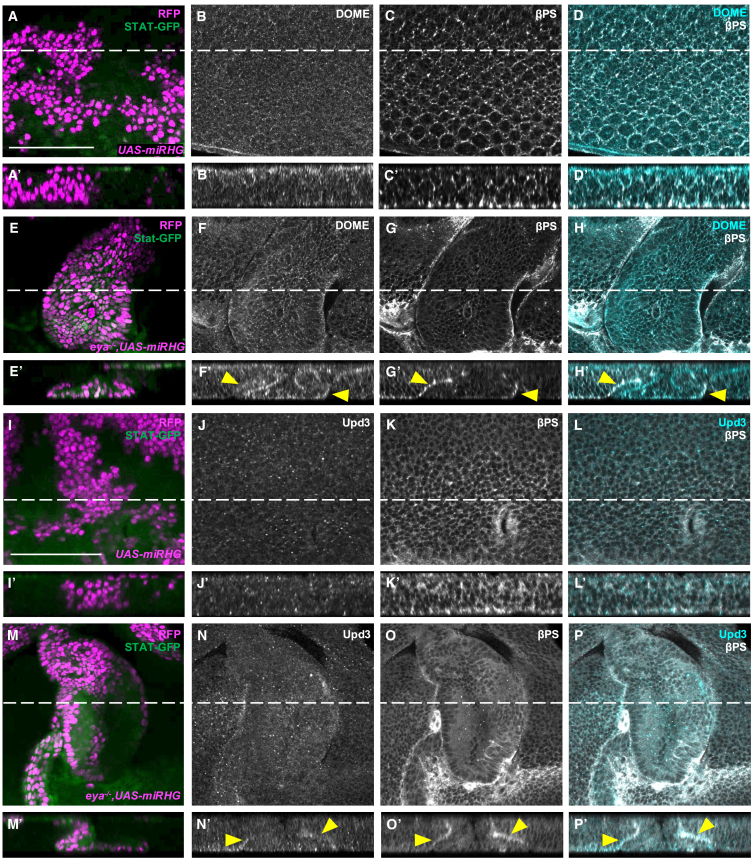
Clones with switched cell fate induce STAT activation via Dome mislocalization (A–H) STAT-GFP expression in eye discs bearing RFP-labeled MARCM clones of UAS-*miRHG* (A), or *eya*^−/−^+UAS-*miRHG* (E), stained with anti-DOME (cyan) and anti-βPS (white). Lines represent the position of lateral section images (A′–D′ for A–D, E′–H′ for E–H). Apical side to the top, basal side to the bottom. Arrowheads indicate overlapping signals. Scale bar: 50 μm (I–P) STAT-GFP expression in eye discs bearing RFP-labeled MARCM clones of UAS-*miRHG* (I), or *eya*^−/−^+UAS-*miRHG* (M), stained with anti-Upd3 (cyan) and anti-βPS (white). Lines represent the position of lateral section images (I′–L′ for I–L, M′–P′ for M–P). Apical side to the top, basal side to the bottom. Arrowheads indicate overlapping signals. Scale bar: 100 μm.

Finally, we sought to understand the mechanism by which cell-fate switch activated JAK-STAT signaling via Upd3. Since Dome is normally localized at the apical surface in the eye epithelium (Figures 5A–5D),^33^ it cannot meet its ligand Upd3 that is present in the basal extracellular region of the epithelium (Figure 4J). Remarkably, *eya*^−/−^ clones exhibited a significant deformation of epithelial structure with an invagination, which was accompanied by partial mislocalization of Dome from the apical surface of the epithelium to the basal membrane, being co-localized with the basal protein integrin βPS (Figures 5E–5H, arrowheads), where Upd3 is present. To further analyze the epithelial structure of *eya*^−/−^ cells, we examined a transmembrane protein PTP10D, which is exclusively localized to the apical membrane.^34^ Notably, Dome and PTP10D showed essentially distinct, separated localizations, with Dome appearing at the basal side (Figures S6A–S6D), suggesting that cell orientation is retained in *eya*^−/−^ clones. Staining with anti-Fasciclin 3 (Fas3), a cell-adhesion molecule used to visualize cell shape, also showed intact cellular structure of *eya*^−/−^ clones (Figures S6E–SH6). Moreover, the partial mislocalization of Dome to the basal membrane was also observed in Ras^V12^/*eya*^−/−^ tumors (Figures S7A–S7H) and in *Abd-B*-overexpressing clones in the eye disc (Figures S7I–S7L). To further validate the interaction of the ligand Upd3 and its receptor Dome, we generated an anti-Upd3 antibody (Figure S8A). Co-immunostaining of Upd3 with integrin showed accumulation of Upd3 at the basal side (Figures 5M–5P), suggesting a possible physical interaction between basal Dome and Upd3.

**Figure 6:**
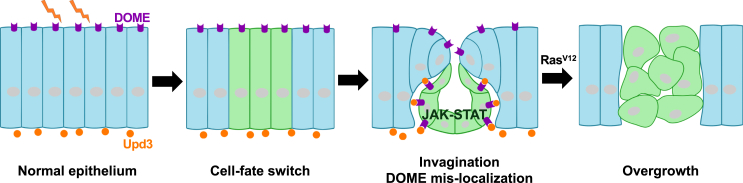
Hypothesis model for the mechanism of cell-fate switch-induced oncogenic cooperation with Ras^V12^. Under normal condition, the ligand Upd3 and its receptor Dome are segregated at the basal and apical sides of the epithelium. Switch in cell fate induces epithelial deformation and allowing interaction of Dome with Upd3, which promotes Ras-induced tumorigenesis.

We further questioned whether epithelial deformation was correlated with JAK-STAT signaling activation. Indeed, overexpression of Rho1^V14^, a constitutively activated form of actin cytoskeleton regulator Rho1, which caused severe deformation of epithelium both inside and outside clones in the imaginal disc,^25^ led to partial mislocalization of Dome to the basal membrane and activation of JAK-STAT signaling in the eye discs (Figures S9A–S9E, compare to Figure 2M, indicated by arrowheads). Interestingly, we found that Rho1^V14^ clones also caused cell-fate switch, as visualized by anti-EYA staining (Figures S9F). Notably, it has been reported that overexpression of Rho1^V14^ promotes Ras^V12^-induced overgrowth.^35^ These data suggest that loss of *eya* causes cell-fate switch and induces epithelial deformation, which is correlated with and may be the driver for JAK-STAT signaling activation. Together, our data suggest that cell-fate switch induces mislocalization of Dome from the apical to the basal epithelial membrane, possibly by epithelial invagination, thereby causing Dome to meet its ligand Upd3 and thus JAK-STAT activation (Figure 6).

## Discussion

Loss of cellular identity, including de-differentiation and *trans*-differentiation, is a hallmark of human cancer.^36^ Canonical *Drosophila* polarity-deficient tumor models also recapitulate loss of cell fate.^4^^,^^37^ Prevalent inflammatory signalings, including JNK and JAK-STAT signaling, are frequently activated in tumors through biochemical modification of key components in the pathways including ligand overexpression or kinase hyperactivation.^20^^,^^21^ Another tumor model with epigenetic perturbation caused by dysfunction of polycomb repressive complex components *polyhomeotic* (Ph) also exhibits tumor mass with a primitive state-like signature, and such tumorigenesis requires JAK-STAT activation caused by upregulation of Upd ligands.^26^^,^^38^^,^^39^ On the other hand, cell-fate switch has been implicated in tumor progression by the evidence that mis-specified cells have higher propensity to become malignant, while restoring developmental cell-fate program overrides neoplastic transformation.^19^^,^^40^ However, little attention has been paid to the exact role and mechanism of cell-fate switch in driving tumorigenesis.^19^ Here, our data substantiated the previously underrated role of cell specification defects in driving tumorigenesis through activation of JAK-STAT signaling. Instead of common biochemical activation of oncogenic signaling, our observations provide a paradigm of structural or physical activation of oncogenic signaling by inducing interaction between oncogenic ligand and receptor through epithelial deformation, which are normally segregated at the basal and apical sides of the epithelium, respectively.

Our study thus highlights the underestimated role of the physical ligand-receptor segregation in preserving epithelial integrity. Notably, the ligand-receptor segregation has been shown to be important in maintaining tissue homeostasis in human airway epithelia. When the epithelium is compromised by mechanical injury or opened tight junction, the apically localized growth factor heregulin activates the receptor erbB2 at the basolateral membrane to restore epithelial integrity.^41^ In addition, a recent study in *Drosophila* has shown that, when epithelial cells lose their apicobasal polarity or the epithelium suffer from physical injury, tumor necrosis factor (TNF) receptor Grindelwald (Grnd) re-localizes from the apical to basal wing epithelium, where the fat body-derived TNF ligand Eiger is present, leading to activation of downstream JNK signaling.^42^ Our data suggest that apically localized Dome interacts with the basally distributed Upd3 when cell-fate switch causes epithelial deformation. As tumor progression inevitably accompanies disruption in cell-fate program and epithelial architecture, such ligand-receptor interaction would provide novel insights of how complex signaling pathways cooperates to drive tumor progression.

Currently it is unclear how loss of *eya* induces invagination and subsequent Dome mislocalization to the basal side. One possible mechanism is that changes in adhesion of mutant cells to neighboring wild-type cells lead to cell sorting and segregation of mutant cells from epithelium, which is a common mechanism during morphogenesis to eliminate mis-specified cells.^25^^,^^43^^,^^44^ At the tissue level, intense apical constriction occurs at clone boundaries between mutant and wild-type cells, inducing invagination and tissue deformation to expel mutant clones. Elevated endocytosis in response to tissue deformation might be a potential mechanism to promote mislocalization of plasma membrane proteins to the region that has more access to ligands.^45^ It is also possible that physical force, which is a major factor driving invagination, could lead to receptor movement within membrane to alter their spatial distribution.^46^^,^^47^ Subsequent studies may identify the roles of adhesion molecules and endocytosis components in the tissue deformation and receptor mislocalization process.

The theory found in this study may also apply for malignant tumorigenesis in humans, as defect in the epithelial structure is a hallmark of human malignant cancers.^9^ Given that signaling molecules identified in this study are all conserved in humans, the tumor progression mechanism via disruption of epithelial architecture could become a potential target of anti-cancer therapy.

### Limitations of the study

Our data propose a proof of concept of receptor mislocalization in tissue deformation driving tumorigenesis. Detailed mechanisms of the receptor mislocalization are needed to be illustrated in the future, which could be regulated by adhesion molecules and the endocytic machinery. It is also important to utilize or generate novel models that could uncouple tissue deformation with mechanical stress response, which is already wildly implicated in cancer. Subsequent research should also address the quantitative understanding of the ligand-receptor interaction within the tissue.

## Resource availability

### Lead contact

Further information and requests for resources and reagents should be directed to and will be fulfilled by the lead contact, Tatsushi Igaki (igaki.tatsushi.4s@kyoto-u.ac.jp).

### Materials availability

*Drosophila* lines generated in this study are available from the lead contact without restriction.

### Data and code availability

• All data reported in this paper will be shared by the lead contact upon request.
• This paper does not report original code.
• Any additional information required to reanalyze the data reported in this paper is available from the lead contact upon request.

## Acknowledgments

We thank K. Baba, M. Tanaka, M. Koijima, M. Matsuoka, Y. Kobe, and K. Gomi for technical support, Yu-Chen Tsai, the Bloomington *Drosophila* Stock Center, the National Institute of Genetics Stock Center (NIG-FLY), the *Drosophila* Genomics and Genetic Resources (DGGR, Kyoto Stock Center) for fly stocks and reagents. We also thank members of the Igaki laboratory for discussions.

This work was supported by grants from the 10.13039/501100001691JSPS KAKENHI (grant number 21K07120) to K.T, the 10.13039/501100001700MEXT/JSPS KAKENHI (grant number 21H05284 and 21H05039) to T.I, 10.13039/100009619AMED-CREST, 10.13039/100009619Japan Agency for Medical Research and Development (grant number 23gm1710002h0002) to T.I, the 10.13039/100007449Takeda Science Foundation to T.I, and the 10.13039/100007428Naito Foundation to T.I. The funders had no role in study design, data collection and analysis, decision to publish, or preparation of the manuscript.

## Author contributions

S.O. and T.I. designed screens; J.L. and W.Y. conducted screens; S.K. and K.S. generated CRISPR-Cas9-mediated knockout fly strains; T.K. and M.M. generated Upd3 antibody; J.L., K.T., S.O., and T.I. designed subsequent experiments; J.L. performed the rest of the experiments; J.L., K.T., S.O., and T.I. analyzed the data; and J.L. and T.I. wrote the manuscript.

## Declaration of interests

The authors declare no competing interests.

## STAR★Methods

### Key resources table

**Table:** 

REAGENT or RESOURCE	SOURCE	IDENTIFIER
**Antibodies**		

Mouse anti-Eya	Developmental Studies Hybridoma Bank	Cat# eya10h6, RRID:AB_528232
Mouse anti-βPS	Developmental Studies Hybridoma Bank	Cat# cf.6g11, RRID:AB_528310
chicken anti-β-galactosidase	Abcam	Cat# ab9361, RRID:AB_307210
Chicken anti-GFP	AVES Labs	Cat# GFP-1010, RRID:AB_2307313
Rat anti-E-cad	Developmental Studies Hybridoma Bank	Cat# DCAD2, RRID:AB_528120
Rabbit anti-aPKC	Santa Cruz Biotechnology	Cat# sc-216, RRID:AB_2300359
Mouse anti-PTP10D	Developmental Studies Hybridoma Bank	Cat# 8b22f5, RRID:AB_528443
Rabbit anti-Dome	Ghiglione et al.^48^	N/A
Rat anti-Crb	Izaddooost et al.^49^	N/A
Rat anti-Upd3	This study	N/A
Alexa Fluor 647 Phalloidin	Thermo Fisher Scientific	Cat#A-22287
Goat anti-mouse Alexa 405	Thermo Fisher Scientific	Cat# A-31553, RRID:AB_221604
Goat anti-mouse Alexa 546	Thermo Fisher Scientific	Cat#A-11030, RRID:AB_2737024
Goat anti-mouse Alexa 647	Thermo Fisher Scientific	Cat# A-32728, RRID:AB_2633277
Goat anti-rabbit Alexa 647	Thermo Fisher Scientific	Cat#A-21245, RRID:AB_2535813
Goat anti-chicken Alexa 647	Thermo Fisher Scientific	Cat# A-21449, RRID:AB_2535866
Goat anti-rat Alexa 647	Thermo Fisher Scientific	Cat# A-21247, RRID:AB_141778
Chemicals		
Schneider’s Drosophila medium	Thermo Fisher Scientific	Cat#21720024
Slow Fade Gold antifade reagent with DAPI	Thermo Fisher Scientific	Cat#S36937

**Experimental models: organisms/strains**		

*Drosophila melanogaster: UAS-miRHG*	Siegrist et al.^50^	N/A
*Drosophila melanogaster: 10*x*STAT92E-GFP*	Bach et al.^51^	N/A
*Drosophila melanogaster: UAS-Luc-RNAi*	Bloomington Drosophila Stock Center	BDSC#31603
*Drosophila melanogaster: UAS-STAT92E-RNAi*	Bloomington Drosophila Stock Center	BDSC#35600
*Drosophila melanogaster: UAS-Dome-RNAi*	Bloomington Drosophila Stock Center	BDSC#28983
*Drosophila melanogaster: UAS-Upd1-RNAi*	Bloomington Drosophila Stock Center	BDSC#33680
*Drosophila melanogaster: UAS-Upd2-RNAi*	National Institute of Genetics	NIG#5988R-1
*Drosophila melanogaster: UAS-Upd3-RNAi*	Bloomington Drosophila Stock Center	BDSC#32859
*Drosophila melanogaster: Upd1-lacZ*	Tsai and Sun^27^	N/A
*Drosophila melanogaster: Upd3-lacZ*	Bunker et al.^52^	N/A
*Drosophila melanogaster: UAS-Upd3-GFP*	Tsai and Sun^27^	N/A
*Drosophila melanogaster: Upd3-Gal4*	Tsai and Sun^27^	N/A
*Drosophila melanogaster: UAS-eya*	Bloomington Drosophila Stock Center	BDSC#5675
*Drosophila melanogaster: UAS-Abd-B*	Kyoto Stock Center	DGGR#106120
*Drosophila melanogaster: UAS-Rho1*^*V14*^	Bloomington Drosophila Stock Center	BDSC#8144
*Drosophila melanogaster: Δupd3*	Bloomington Drosophila Stock Center	BDSC#55728
*Drosophila melanogaster: UAS-vg*	Bloomington Drosophila Stock Center	BDSC#37296
*Drosophila melanogaster: UAS-ey*	Bloomington Drosophila Stock Center	BDSC#6294
*Drosophila melanogaster: eya*^*SK5*^	National Institute of Genetics	NIG#M2L-2129
*Drosophila melanogaster: eya*^*SK7*^	National Institute of Genetics	NIG#M2L-2130

**Software and algorithms**		

Leica LAS AF software	Leica Microsystems	http://www.leica-microsystems.com/
ImageJ software	National Institute of Health	https://imagej.nih.gov/ij/
Excel	Microsoft	https://products.office.com/en-gb/excel
Graph Pad Prism	Graph Pad	https://www.graphpad.com/

**Other**		

Leica TCS SP8 microscope	Leica Microsystems	https://www.leica-microsystems.com/

### Experimental model and subject details

#### Fly strains and generation of MARCM clones

Fluorescently labeled mitotic clones were produced in larval imaginal discs using the following

strains: w;Tub-Gal80,FRT40A,UAS-Ras^V12^;eyFLP6,Act>y^+^>Gal4,UAS-GFP (Ras^V12^ tester); eyFLP1,UAS-Dcr2;Tub-Gal80,FRT40A;Act>y^+^>Gal4,UAS-GFP (40A tester); UbxFLP;Tub-Gal80,FRT40A;Act>y^+^>Gal4,UAS-GFP (40A wing tester) Tub-Gal80,FRT40A; eyFLP6,Act>y^+^>Gal4,UAS-His2Am.RFP (RFP 40A tester); Tub-Gal80,FRT19A;eyFLP5, Act>y^+^>Gal4,UAS-GFP;+ (19A tester); Tub-Gal80,FRT19A;+;eyFLP6,Act>y^+^>Gal4,UAS-His2A.mRFP (RFP 19A tester). Principle of MARCM clone generation was described previously.^13^ Additional strains used are the following: CRISPR-Cas9-mediated knockout fly library,^53^ UAS-miRHG (Dr. Hariharan), 10xSTAT92E-GFP (Dr. Bach), UAS-LUC-RNAi (BDSC31603), UAS-Stat92E-RNAi (BDSC35600), UAS-Dome-RNAi (BDSC28983), UAS-Upd1-RNAi (BDSC33680),^54^ UAS-Upd2-RNAi (NIG5988R-1),^55^ UAS-Upd3-RNAi (BDSC32859),^56^ Upd1-lacZ (Dr. Sun), Upd3-lacZ (Dr. Bilder), UAS-Upd3-GFP, Upd3-Gal4 (Dr. Sun), *Δupd3* (BDSC55728), UAS-*Abd-B* (DGGR10612), UAS-*eya* (BDSC5675), UAS-*vg* (BDSC37296), UAS-*ey* (BDSC6294), UAS-Rho1^V14^ (BDSC8144). Detailed genotypes are listed in Table S2.

### Method details

#### Generation of CRISPR-Cas9-mediated knockout fly library

A large-scale mutant library covering approximately 70% of the genes (approximately 2,500 protein-coding genes) on chromosome arm 2L, including *eya*^*SK5*^, *eya*^*SK7*^ that were made by the transgenic CRISPR/Cas9 technique.^53^ For each gene, eight independent prospective mutant lines were molecularly characterized by direct sequencing of PCR products. Two lines carrying frameshift mutations, if available, were selected as null mutants and were subjected to further analysis. The molecular details of the mutant alleles *eya*^*SK5*^, *eya*^*SK7*^ are shown in Table S1.

#### Generation of monoclonal Upd3 antibody

His-tagged (for antigen production) or MBP-tagged (for monoclonal antibody screening) 75-401aa of Upd3 protein was expressed in BL21-CodonPlus-RP (Agilent Technologies, Santa Clara, CA) transformed with pET-28a (Invitrogen) or pMAL (New England Biolabs, Beverly, MA), respectively. Each His or MBP fusion protein was purified through affinity chromatography with TALON metal affinity resin (Clonetech, Palo Alto, CA) or amylose resin (New England Biolabs), respectively. We produced a rat monoclonal antibody against Upd3 as described previously.^57^ Briefly, the Upd3 antigen emulsion was injected into WKY/NCrl rats. The treated rats were euthanized 21 days after the injection, and lymphocytes were fused with SP2/0-Ag14 myeloma cells. After the cell fusion, culture supernatants were screened to confirm positive clones by a solid-phase enzyme-linked immunosorbent assay (ELISA).

#### Immunostaining

Wandering 3rd instar larvae were dissected in PBS under binocular stereomicroscopes and fixed with 4% paraformaldehyde (PFA) in PBS. PBT (PBS with 1%Triton-X) was used as washing solution and PBTn (PBT with 5% donkey serum) was used as blocking agent. Larval tissues were stained with standard immunochemical procedures using mouse anti-βPS (1:100), mouse anti-Eya (1:50), chicken anti-β-galactosidase antibody (1:1000), rabbit anti-Dome (1:200), rat anti-Upd3 (1:10), mouse anti-PTP10D (1:200), rat anti-Crb (1:500), rabbit anti-aPCK (1:500), Phalloidin (1:50). Secondary antibodies used are as follows: Goat anti-mouse Alexa 405, 546, 647, Goat anti-rabbit Alexa 647, Goat anti-chicken Alexa 647, Goat anti-rat Alexa 647 (1:250). For extracellular GFP staining, larvae were dissected in Schneider’s *Drosophila* medium with 5% FBS and incubated with anti-chicken GFP antibodies (1:20) or anti-rat Upd3 (1:5) for 1 hour. Samples were then washed with ice-cold PBS for 15min, 3 times. Standard procedures were performed for fixation and secondary antibody labeling using PBS.

### Quantification and statistical analysis

Imaginal disc images were taken with TCS-SP8 confocal laser scanning microscope (Leica), respectively. Clone size was measured as GFP positive area/disc area using ImageJ (Fiji) software. Clone STAT-GFP intensity was quantified and normalized with background intensity. One-way ANOVA and unpaired t-tests (GraphPad Prism) were performed significance of difference was represented by p-values (where NS: non-significant difference, ∗p < 0.1, ∗∗p < 0.01, ∗∗∗p < 0.001). All data in bar graphs were expressed as mean ± s.d.

## Supplemental information

Document S1. Figures S1–S9 and Table S1

Table S2. Detailed genotypes shown in Figure 1–5 and S1–S9, related to STAR Methods
