# Supplementary material for: Epithelial cell-fate switch triggering ectopic ligand-receptor-mediated JAK-STAT signaling promotes tumorigenesis in Drosophila
Source: iScience. 2025 Mar 10;28(4):112191. doi: 10.1016/j.isci.2025.112191 (PMC11995115; doi:10.1016/j.isci.2025.112191)
Supplement: Document S1. Figures S1–S9 and Table S1 [file mmc1.pdf]

iScience, Volume *28*

Supplemental information

Epithelial cell-fate switch triggering ectopic ligand-receptor-mediated JAK-STAT signaling promotes tumorigenesis in *Drosophila*

Jiaqi Li, Kiichiro Taniguchi, Weiran Ye, Shu Kondo, Tomoe Kobayashi, Makoto Matsuyama, Kuniaki Saito, Shizue Ohsawa, and Tatsushi Igaki

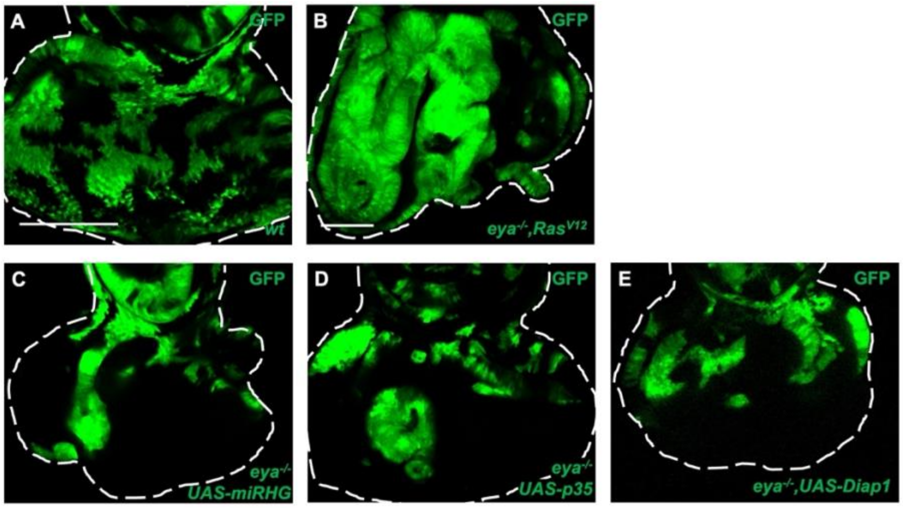

Fig. S1 Inhibition of apoptosis fails to induce overgrowth of *eya*$^{-/-}$ clone. Related to Fig. 1.

(A-E) Eye discs bearing GFP-labeled MARCM clones of wild-type (A), Ras^{V12}+*eya*$^{-/-}$ (B), *eya*$^{-/-}$ +UAS-miRHG (C), *eya*$^{-/-}$+UAS-*p35* (D), or *eya*$^{-/-}$+UAS-*Diap1* (E). Scale bar: 100 µm, Scale bar in (A) is applicable for (C-E).

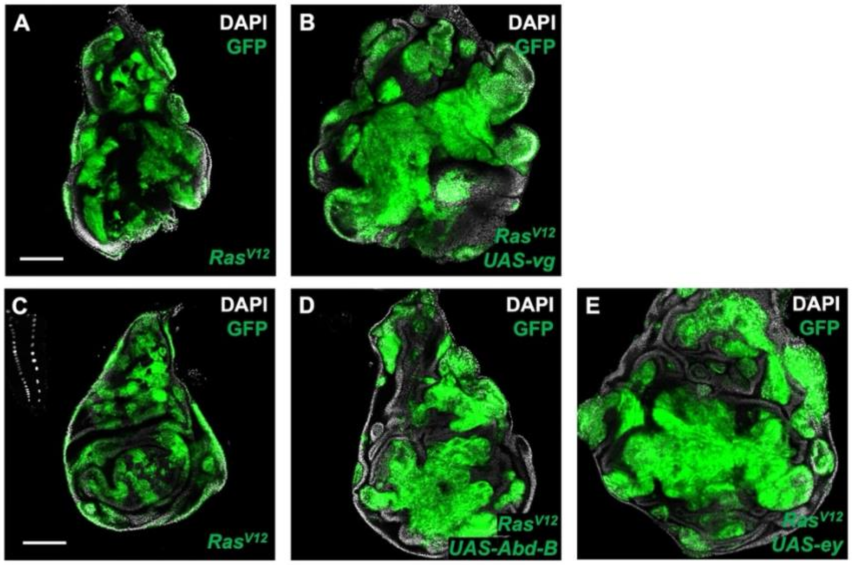

Fig. S2 Multiple selector genes show oncogenic cooperation with Ras^{V12}. Related to Fig. 1.

(A and B) Eye discs bearing GFP-labeled MARCM clones of Ras^{V12} (A), or Ras^{V12}+UAS-*vg* (B). Scale bar: 100 μm.

(C-E) Wing disc bearing GFP-labeled MARCM clones of Ras^{V12} (C), Ras^{V12}+ UAS-*Abd-B* (D), or Ras^{V12}+ UAS-*ey* (E). Scale bar: 100 μm.

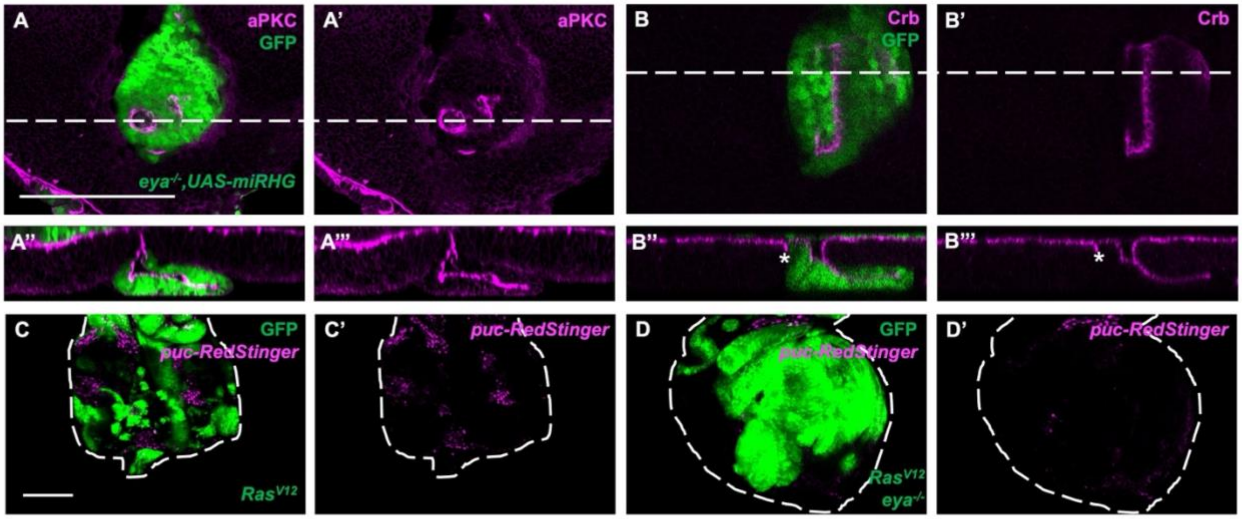

Fig. S3 JNK signaling is not involved in Ras^{V12}*eya*$^{-/-}$ overgrowth. Related to Fig. 2.

(A and B) Eye disc bearing GFP-labeled MARCM clones of *eya*$^{-/-}$+UAS-*miRHG*, stained with anti-aPKC (A, magenta) and anti-Crb (B, magenta). Lines represent the position of lateral section images (A'' and A''' for A, B'' and B''' for B). Asterisk indicates new invagination surface at clone boundary. Apical side to the top, basal side to the bottom. Scale bar: 100 µm.

(C and D) *puc-stinger* expression in eye discs bearing GFP-labeled MARCM clones of Ras^{V12} (C), or Ras^{V12}+*eya*$^{-/-}$ (D). Scale bar: 100 µm.

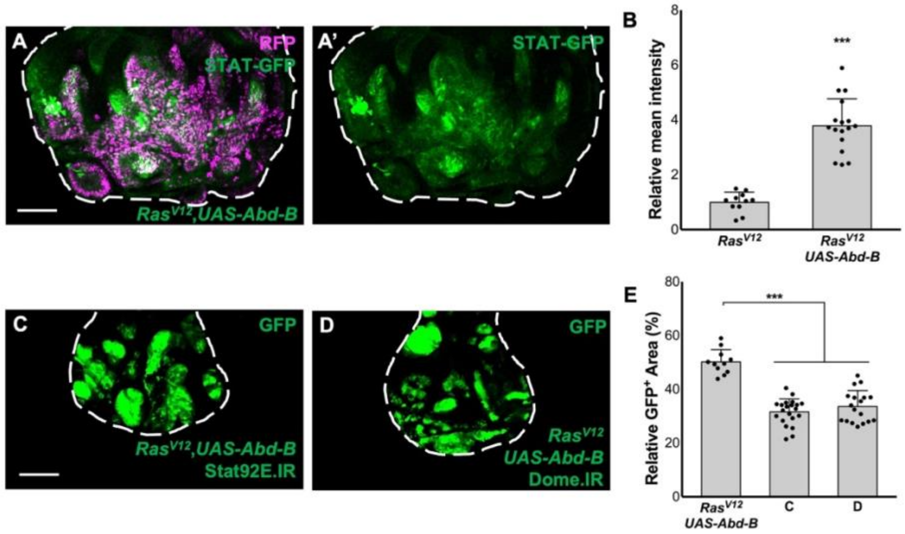

Fig. S4 Cell-fate switch induced by selector gene overexpression upregulate STAT activity. Related to Fig. 2.

(A) STAT-GFP expression in eye discs bearing RFP-labeled MARCM clones of Ras^{V12}+UAS-*Abd-B*. Scale bar: 100 μm.

(B) Quantification of STAT-GFP intensity for (A), compared with Ras^{V12} in Fig. 2 (average of Ras^{V12} is set to 1) ($n > 10$, number of eye discs). $***p < 0.001$; Mann-Whitney test. Data are represented as mean ± s.d.

(C-D) Eye discs bearing GFP-labeled MARCM clones of Ras^{V12}+UAS-*Abd-B*+Stat92E.IR (C), or Ras^{V12}+UAS-*Abd-B*+Dome.IR (D). Scale bar: 100 μm.

(E) Quantification of relative GFP clone size for (C and D) ($n > 10$, number of eye discs). $***p < 0.001$; Kruskal-Wallis test. Data are represented as mean ± s.d.

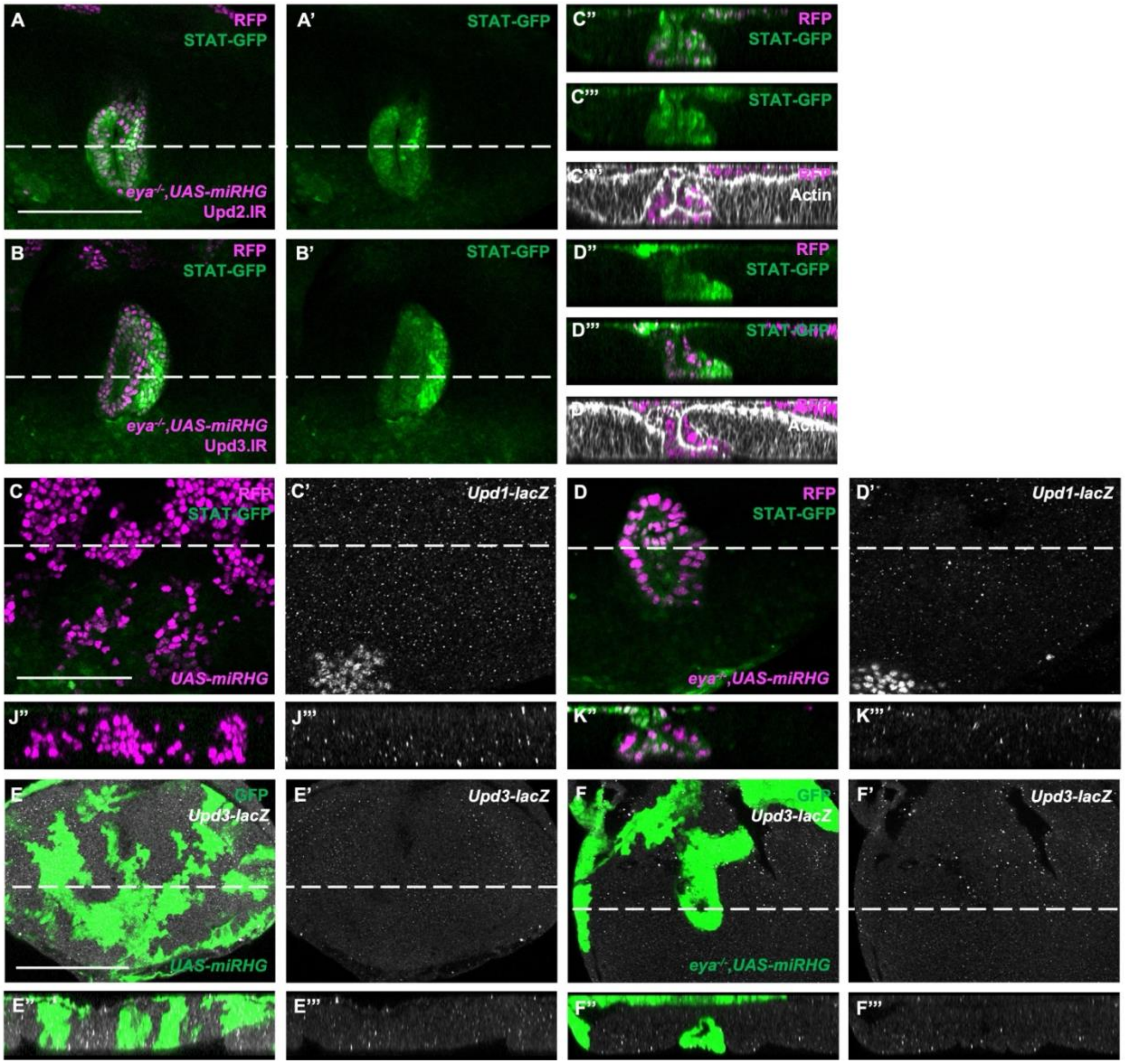

Fig. S5 Upd is not upregulated and involved in STAT activation in *eya* mutant clones. Related to Fig. 3.

(A and B) STAT-GFP expression in eye discs bearing RFP-labeled MARCM clones of *eya*$^{-/-}$ +UAS-*miRHG*+Upd2.IR (A), or *eya*$^{-/-}$+UAS-*miRHG*+Upd3.IR (B), stained with Phalloidin (white). Lines represent the position of lateral section images (A''-A'''' for A, B''-B'''' for B). Apical side to the top, basal side to the bottom. Scale bar: 100 μm.

(C and D) *Upd1-lacZ/+* eye- discs bearing RFP-labeled MARCM clones of UAS-*miRHG* (C), or *eya*$^{-/-}$+UAS-*miRHG* (D), stained with anti-β-galactosidase (white). Lines represent the position of lateral section images (C''-C''' for C, D''-D''' for D). Apical side to the top, basal side to the bottom. Scale bar: 50 μm.

(E and F) *Upd3-lacZ/+* eye- discs bearing GFP-labeled MARCM clones of UAS-*miRHG* (E), or *eya*$^{-/-}$+UAS-*miRHG* (F), stained with anti-β-galactosidase (white). Lines represent the position of lateral section images (E''-E''' for E, F''-F''' for F). Apical side to the top, basal side to the bottom. Scale bar: 100 μm.

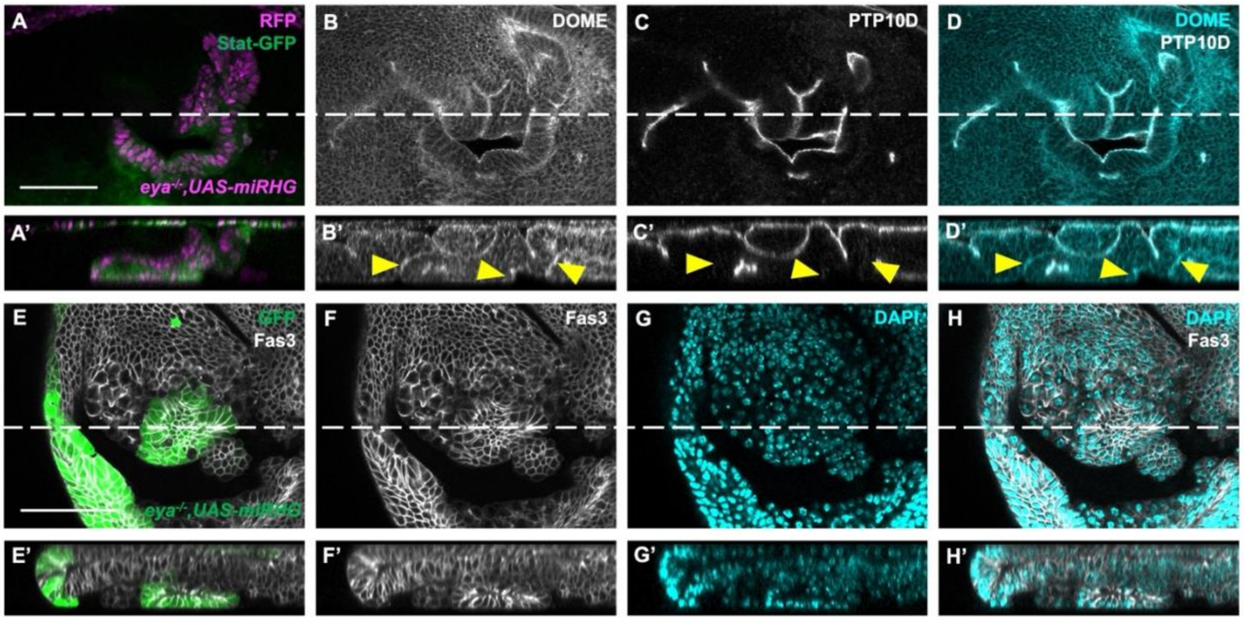

Fig. S6 Clones with switched cell fate retain proper cell orientation and structure. Related to Fig. 5.

(A-D) STAT-GFP expression in eye disc bearing RFP-labeled MARCM clones of *eya*$^{-/-}$+UAS-*miRHG*, stained with anti-PTP10D (white) and anti-DOME (cyan). Lines represent the position of lateral section images (A'-D' for A-D). Apical side to the top, basal side to the bottom. Arrowheads indicate non-overlapping signals. Scale bar: 100 µm.

(E-H) Eye disc bearing GFP-labeled MARCM clones of *eya*$^{-/-}$+UAS-*miRHG,* stained with anti-Fas3 (white) and DAPI (cyan). Lines represent the position of lateral section images (E'-H' for E-H). Apical side to the top, basal side to the bottom. Scale bar: 100 µm.

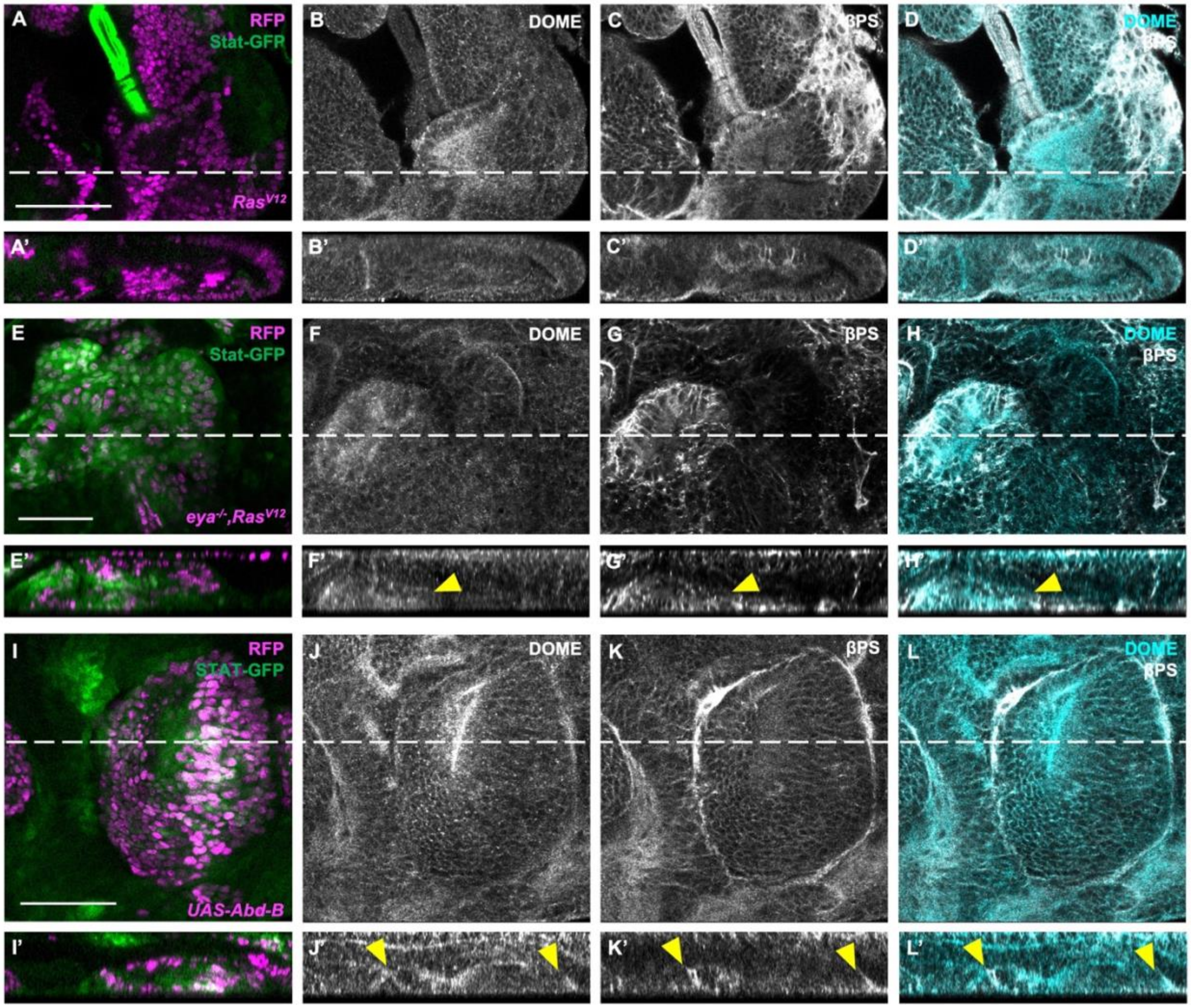

Fig. S7 Clones with switched cell fate upregulate STAT through Dome mislocalization. Related to Fig. 5.

(A-H) STAT-GFP expression in eye discs bearing RFP-labeled MARCM clones of Ras^{V12} (A), or Ras^{V12}+$eya^{-/-}$ (E), stained with anti-βPS (white) and anti-DOME (cyan). Lines represent the position of lateral section images (A'-D' for A-D, E'-H' for E-H). Apical side to the top, basal side to the bottom. Arrowheads indicate overlapping signals. Scale bar: 100 μm.

(I-L) STAT-GFP expression in eye discs bearing RFP-labeled MARCM clones of UAS-*Abd-B*, stained with anti-βPS (white) and anti-DOME (cyan). Lines represent the position of lateral section

images (I'-L' for I-L). Apical side to the top, basal side to the bottom. Arrowheads indicate overlapping signals. Scale bar: 50 μm.

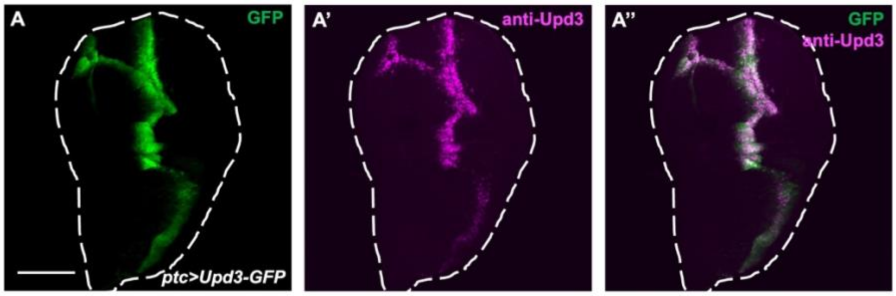

Fig. S8 Validation of the anti-Upd3 antibody. Related to Fig. 5.

(A) Wing disc with *ptc*-Gal4 driving UAS-Upd3-GFP, stained with anti-Upd3 (magenta). Scale bar: 100 μm.

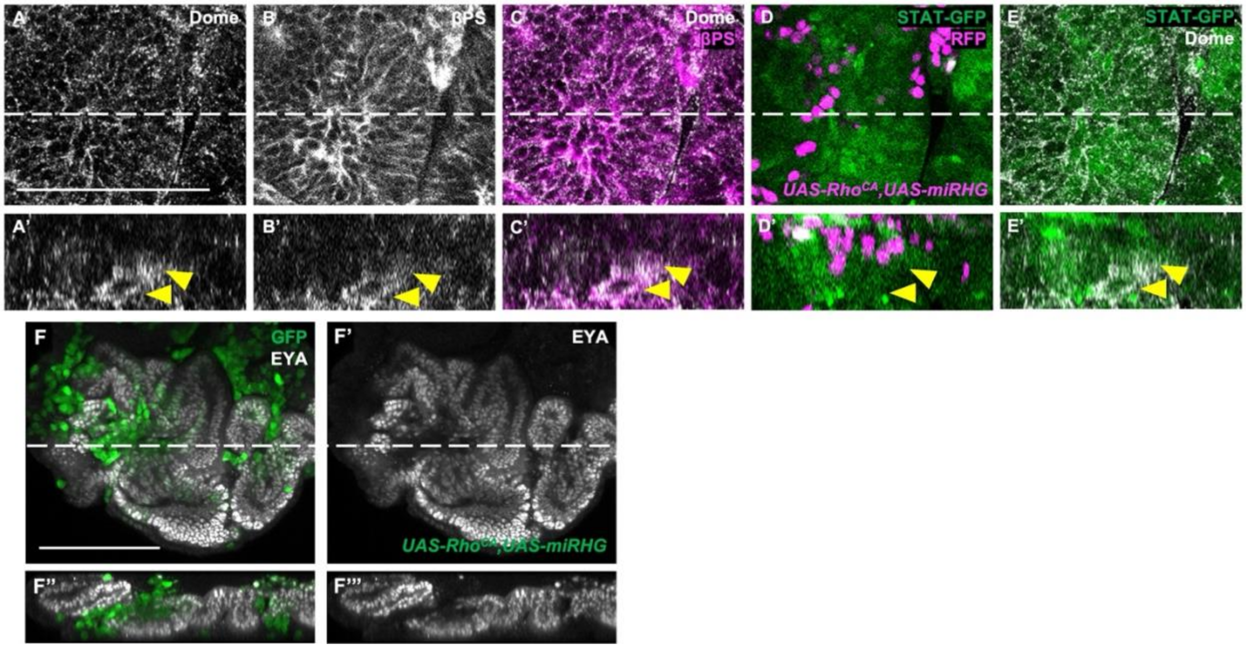

Fig. S9 Rho1^{V14} overexpression shows Dome mislocalization and activation of JAK-STAT. Related to Fig. 5.

(A-E) STAT-GFP expression in eye discs bearing RFP-labeled MARCM clones of UAS-*miRHG*+UAS-*Rho1*^{V14}, stained with anti-βPS (magenta) and anti-DOME (white). Lines represent the position of lateral section images (A'-E' for A-E). Apical side to the top, basal side to the bottom. Arrowheads indicate overlapping signals. Scale bar: 100 μm.

(F) Eye disc bearing GFP-labeled MARCM clones of UAS-*miRHG*+UAS-*Rho1*^{V14}, stained with anti-EYA (white). Lines represent the position of lateral section images (F'' and F''' for F). Apical side to the top, basal side to the bottom. Scale bar: 100 μm.

Table S1 The molecular details of the CRISPR-Cas9 mutant alleles. Related to Fig. 1.

Allele name	Deleted position (base)	5'-3' sequence (underline: deleted bases)
eya^{SK5}	686-696	GGCCG(<u>GATCCAATTTG</u>)TACGG
eya^{SK7}	688-715	CCGGA(<u>TCCAATTTGTACGGCTGCAGCTCGGCCA</u>)GCAAT
